# Association of Early Nutritional Status With Child Development in the Asia Pacific Region

**DOI:** 10.1001/jamanetworkopen.2021.39543

**Published:** 2021-12-16

**Authors:** Frederick K. Ho, Nirmala Rao, Keith T. S. Tung, Rosa S. Wong, Wilfred H. S. Wong, Joanna Y. L. Tung, Gilbert T. Chua, Winnie W. Y. Tso, John Bacon-Shone, Ian C. K. Wong, Aisha Yousafzai, Charlotte Wright, Patrick Ip

**Affiliations:** 1 Institute of Health and Wellbeing, University of Glasgow, Glasgow, United Kingdom; 2 Faculty of Education, The University of Hong Kong, Hong Kong; 3 Department of Paediatrics and Adolescent Medicine, Queen Mary Hospital, The University of Hong Kong, Hong Kong; 4 Department of Paediatrics, Hong Kong Children’s Hospital, Hong Kong; 5 The State Key Laboratory of Brain and Cognitive Sciences, University of Hong Kong, Hong Kong; 6 Social Science Research Centre, The University of Hong Kong, Hong Kong; 7 Centre for Safe Medication Practice and Research, The University of Hong Kong, Hong Kong; 8 UCL School of Pharmacy, University College London, London, United Kingdom; 9 Department of Global Health and Population, Harvard University, Cambridge, Massachusetts; 10 School of Medicine, Dentistry, and Nursing, University of Glasgow, Glasgow, United Kingdom

## Abstract

**Importance:**

Stunting was used as a proxy for underdevelopment in early childhood in previous studies, but the associations between child development and other growth and body composition parameters were rarely studied.

**Objective:**

To estimate the association between malnutrition and early child development (ECD) at an individual level.

**Design, Setting, and Participants:**

This population-based, cross-sectional study used data from the East Asia Pacific Early Child Development Scales, a population-representative survey of children aged 3 to 5 years old, conducted in 2012 to 2014 in communities in Cambodia, China, Mongolia, Papua New Guinea, and Vanuatu. Data analysis was performed from November 2019 to April 2021.

**Exposures:**

Stunting (height-for-age [HFA] *z* score less than −2), wasting (weight-for-height *z* score less than −2), overweight (weight-for-height *z* score greater than 2), body mass index (BMI)–for-age *z* score, and body fat proportion based on existing growth standard and formula.

**Main Outcomes and Measures:**

ECD directly assessed using the validated East Asia–Pacific ECD Scales.

**Results:**

A total of 7108 children (3547 girls; mean [SD], age 4.48 [0.84] years) were included in this study. The prevalence of stunting was 27.1% (range across countries, 1.2%-55.0%), that of wasting was 13.7% (range, 5.4%-35.9%), and that of overweight was 15.9% (range, 2.2%-53.7%). Adjusted for country variations, age, sex, urbanicity, family socioeconomic status, and body fat proportion, ECD was linearly associated with HFA (β, 1.57; 95% CI, 1.35-1.80) and BMI-for-age (β, 0.64; 95% CI, 0.45-0.82). After adjustment for BMI and height, better ECD was associated with low body fat proportion (β, 0.93; 95% CI, 0.45-1.42). The association of HFA was more pronounced in Southeast Asia and the Pacific region than in East Asia, and the association of fat proportion was specific to children living in urban environments.

**Conclusions and Relevance:**

HFA, BMI-for-age, and body fat proportion were independently associated with ECD, and these findings suggest that future studies should consider using these parameters to estimate the prevalence of child underdevelopment; nutritional trials should examine to what extent the associations are causal.

## Key Points

**Question:**

Is early childhood development associated with nutritional and body composition indicators?

**Findings:**

In this cross-sectional study of 7108 children, better early child development was significantly and linearly associated with height-for-age and low body mass index–for-age. After adjustment for body mass index and height, better development was associated with low mid–upper arm fat proportion but not with fat area.

**Meaning:**

These findings suggest that future studies should consider using height-for-age, body mass index–for-age, and body fat proportion to estimate the prevalence of child underdevelopment, and nutritional trials should examine to what extent the associations are causal.

## Teaser

This cross-sectional study estimates the association between malnutrition and early child development at an individual level among children in 5 countries in the Asia Pacific region.

## Introduction

Malnutrition is a global health problem associated with more than 2 million deaths and one-fifth of disability-adjusted life-years among young children.^1^ Nutritional status of children is typically inferred by their growth, including height-for-age (HFA) and weight-for-height (WFH).^2^ Although there have been marked improvements in the past decade, 23% of children were still stunted (defined as an HFA *z* score less than −2) and 8% were wasted (defined as a WFH *z* score less than −2) as of 2017.^3^

Growth restriction often cooccurs with early childhood development (ECD).^4^ The association between HFA and ECD has been well documented in a meta-analysis,^5^ although the evidence was generally weak. Many studies were small and nonrepresentative, which could induce selection bias.^6^ Some did not measure ECD using culturally appropriate direct assessments, casting doubt on its validity.^7^ More importantly, the evidence on other relevant indicators,^8^ such as body weight and fat, were scarce.

Given the dearth of data, disaggregated by age, sex, and urbanicity, on nutritional status of children in many countries in the East Asia–Pacific Region (EAPR), we conducted this study to provide population-representative data on the nutritional status of young children in 5 countries in the EAPR. In addition, because previous studies on the association of nutritional status with ECD often focused only on stunting or HFA in isolation, we also investigated the associations of a comprehensive set of nutritional status and body composition indicators with ECD. Because malnutrition prevalence varies by region and urbanicity, the associations stratified by these 2 factors were also explored.

## Methods

### Study Design and Sampling

This cross-sectional study has been approved by the human research ethics committee of the University of Hong Kong. Written informed consent was obtained from all participating parents. This report follows the Strengthening the Reporting of Observational Studies in Epidemiology (STROBE) reporting guideline.

This study is a part of the East Asia Pacific Early Child Development Scales (EAP-ECDS), a population-representative survey of children aged 3 to 5 years conducted in 2012 to 2014 in 6 countries in the EAPR: Cambodia, China, Mongolia, Papua New Guinea, Timor-Leste, and Vanuatu. The full report of the study has been published elsewhere.^9^ Data from Timor-Leste were not included in this study because the assessors expressed concerns regarding the accuracy of the body weight measurements. No similar feedback was received in other countries.

Multilevel stratified random sampling was used to select a representative sample from each of the participating countries. The sampling frame was determined with collaboration of the national statistics departments of all countries except China, for which the sampling was assisted by the collaborating institutions. Five provinces and municipalities of China (Guizhou, Heilongjiang, Jiangsu, Shanghai, and Zhejiang) were selected to represent a wide spectrum of economic development. Children with special educational needs were excluded from the study. Only 1 child from each family was recruited to avoid interdependence between data points.

### Measurements

#### Anthropometry, Nutritional Status, and Body Composition

Children’s body height, weight, mid–upper arm circumference (AC), and triceps skinfold (TS) thickness were measured with a protocol used in the World Health Organization (WHO) Multicenter Growth Reference Study.^10^ Briefly, the assessors attended multiple sessions of training for measuring the anthropometric parameters, in which the study anthropometrists demonstrated the standard methods to measure all parameters. The assessors also had hands-on measurement trials under the anthropometrists’ observation. Assessors who were found to have substandard procedures or errors were corrected and retrained. Body height was measured to the nearest 1 mm, and weight was measured to the nearest 0.1 kg using standard stadiometer and scale. Mid–upper AC and TS thickness were measured using standard tape measures and calipers to the nearest 1 mm.

These anthropometric parameters were standardized to age- and sex-specific *z* scores based on the WHO Growth Standard.^11^ These *z* scores were then used to define stunting (HFA *z* score less than −2), wasting (WFH *z* score less than −2), and overweight (WFH *z* score greater than 2).^2^ In addition, AC and TS were used to estimate the children’s upper-arm fat area, lean area (including bone and muscle), and fat proportion using a published formula^12^ that has been validated against magnetic resonance imaging measurements. Body mass index (BMI) was calculated as body weight in kilograms divided by height in meters squared, and BMI-for-age *z* score was used in our analyses.

Nutritional status variables (HFA, WFH, BMI*-*for age, mid–upper AC, and TS) were age- and sex-standardized on the basis of the WHO LMS (skew, median, and SD) parameters.^11^ Body composition variables (mid–upper arm fat area, lean area, and fat proportion) were age- and sex-standardized using the current sample. Low HFA (and thus stunting) is regarded as an indicator of chronic malnutrition, whereas low WFH (and thus wasting) and BMI-for-age are regarded as indicators of acute malnutrition. Although the WHO did not specify BMI-for-age to be an indicator of acute malnutrition, WFH and BMI-for-age are conceptually similar indicators: the former is body weight adjusted for height, assuming a uniform distribution across ages, whereas the latter is body weight adjusted for height squared and age. Both indicate normalized body weight.

#### Early Child Development

ECD was measured by the EAP-ECDS, an item-response inventory specifically designed for the participating countries. Details of the theoretical framework, development, validation, and adoption of the scales can be found in previous publications.^13,14^ In brief, the assessment was direct observation of skills using a structured checklist conducted by trained assessors. The scales were developed according to the country-specific goals and values for children. The EAP-ECDS has a total of 85 items measuring the overall developmental status and 7 subscales for measuring Cognitive Development (21 items), Language/Emergent Literacy (16 items), Socioemotional Development (15 items), Motor Development (7 items), Cultural Knowledge and Participation (10 items), Healthy Hygiene and Safety (9 items), and Approaches to Learning (7 items). The scales showed good differentiating power against children’s age and family background. In this study, Total Development was calculated as the unweighted average of all domains in the EAP-ECDS. All the subscales achieved good internal consistency (Cronbach α, 0.84-0.94) in all countries except for the motor development subscale, which had acceptable consistency (Cronbach α, 0.74-0.87). In this study, we focused on the Total, Cognitive, Language/Emergent Literacy, Socioemotional, and Motor Development subscales, and scores were standardized for every 6 months of age (eg, 36 to <42 months, 42 to <48 months, and so forth) so that the age-specific mean (SD) score was 100 (15). This standardization allows comparison of effect size across different subscales.

#### Socioeconomic Status

A composite index was constructed for measuring the multidimensional family socioeconomic status (SES) in this study. The SES index was the first eigenvalue of the principal component analysis results using the correlation matrix from paternal education level, maternal education level, and family assets, including electricity, radio, television, refrigerator, watch, mobile phone, bicycle, animal-drawn cart, agricultural land, livestock, and so forth. The method has been shown to be valid and reliable for representing the overall SES.^15^

### Statistical Analysis

Descriptive statistics were calculated for key parameters. Urban vs rural differences in these variables were quantified by independent *t* tests (continuous variables) and Fisher exact tests (categorical variables). Country prevalence of nutritional status was estimated with country-specific weighting on age, sex, and urbanicity.^16^ The weights were generated from population censuses and were assumed to be deterministic. The associations between nutritional status, body composition, and development were tested using linear additive mixed models.^17^ It was hypothesized, a priori, that optimal nutritional status and body composition were associated with better development. Development scores were the dependent variables, and nutritional status and body composition fitted on penalized splines were the independent variables. Penalized splines were used to avoid assuming linear relationships. We used generalized cross-validation to select the number of knots in the penalized splines, which is robust against knot-related biases.^18^ Nonlinearity was tested using likelihood ratio test comparing a model with the exposure fitted on a spline with a model assuming a linear exposure-outcome association. Child’s age, sex, urbanicity, and family SES index were adjusted as confounders. Country- and province-level variations were modeled as random intercepts to address intracountry and province correlations. Because of the small number of countries, a sensitivity analysis was conducted to include country as fixed factor. Stratified analyses were conducted by region (East Asia [China and Mongolia] vs Southeast Asia and Pacific [Cambodia, Papua New Guinea, and Vanuatu]) and urbanicity (urban vs rural) to examine whether the associations were consistent across these subgroups. Likelihood ratio tests were used to test for any interactions. The associations between development and nutritional status variables were first tested individually. A fully adjusted model was then used to examine independent associations after adjustment of other included nutritional status and body composition indicators. Because of multicollinearity, 1 variable was chosen from each of the following sets based on *R*^2^: weight-for-age and BMI-for-age; and mid–upper AC, TS thickness, lean area, and fat area. Body weight (as indicated by weight-for-age or BMI-for-age) and fat proportion were mutually adjusted so that the former indicated mainly lean mass. To avoid inflating type I error, *P* values were corrected using the Holm Bonferroni procedure, which controls the familywise error rates to the .05 significance level. There were negligible (<5%) missing data in anthropometry measurements and those children were excluded. All tests conducted were 2-sided, and analysis was conducted using R statistical software version 3.23 (R Project for Statistical Computing) with the package gamm4. Data analysis was performed from November 2019 to April 2021.

## Results

This study included 7108 young children (3547 girls; mean [SD] age, 4.48 [0.84] years) from the 5 participating countries (Table 1). Most of the mothers (2059 women [29.97%]) had only completed primary school but there were also 985 mothers (13.86%) who had a bachelor’s degree. The prevalence of stunting of this sample was 27.1% (range across countries, 1.2%-55.0%), that of wasting was 13.7% (range, 5.4%-35.9%), and that of overweight was 15.9% (range, 2.2%-53.7%).

**Table 1.** Characteristics of Participating Children From Cambodia, China, Mongolia, Papua New Guinea, and Vanuatu

Characteristic	Children, No. (%)			*P* value^a^
	All (N = 7108)	Rural (n = 4280)	Urban (n = 2828)	
Age, mean (SD), y	4.48 (0.84)	4.49 (0.84)	4.48 (0.85)	.66
Sex				
Female	3547 (49.90)	2100 (49.07)	1447 (51.17)	.17
Male	3561 (50.10)	2180 (50.93)	1381 (48.83)	
Country				
China	1784 (25.10)	923 (21.57)	861 (30.45)	<.001
Cambodia	1500 (21.10)	852 (19.91)	648 (22.91)	
Mongolia	1247 (17.54)	622 (14.53)	625 (22.10)	
Papua New Guinea	1795 (25.25)	1187 (27.73)	608 (21.50)	
Vanuatu	782 (11.00)	696 (16.26)	86 (3.04)	
Maternal education level				
No formal education	666 (9.37)	580 (13.55)	86 (3.04)	<.001
Primary	2059 (28.97)	1539 (35.96)	520 (18.39)	
Lower secondary	1418 (19.95)	832 (19.44)	586 (20.72)	
Upper secondary	1216 (17.11)	641 (14.98)	575 (20.33)	
Postsecondary	764 (10.75)	361 (8.43)	403 (14.25)	
Bachelor’s degree or above	985 (13.86)	327 (7.64)	658 (23.27)	
Growth indicators				
Stunting	1918 (27.14)	1435 (33.74)	483 (17.16)	<.001
Wasting	946 (13.67)	602 (14.37)	344 (12.61)	.12
Overweight	1098 (15.87)	775 (18.50)	323 (11.84)	<.001
Height-for-age, *z* score, mean (SD)	−0.99 (1.77)	−1.29 (1.79)	−0.54 (1.63)	<.001
Weight-for-height, *z* score, mean (SD)	0.09 (2.15)	0.18 (2.26)	−0.05 (1.94)	<.001
Body mass index–for-age, *z* score, mean (SD)^b^	0.15 (2.14)	0.25 (2.25)	0.00 (1.96)	<.001
Mid–upper arm circumference–for-age	−0.13 (1.77)	−0.27 (1.79)	0.03 (1.73)	<.001
Triceps skinfold thickness–for-age	−0.84 (2.05)	−1.02 (2.06)	−0.57 (1.99)	<.001
Body composition, mean (SD)				
Mid–upper arm lean area, cm^2^	15.09 (5.09)	14.77 (4.68)	15.44 (5.50)	<.001
Mid–upper arm fat area, cm^2^	6.70 (4.21)	6.50 (4.47)	6.92 (3.88)	.001
Mid–upper arm fat proportion	0.29 (0.13)	0.29 (0.14)	0.30 (0.12)	.06
East Asia Pacific Early Child Development Scales standard score, mean (SD)				
Cognitive Development	101.17 (15.28)	99.98 (14.96)	102.96 (15.58)	<.001
Language/Emergent Literacy	101.66 (15.07)	99.51 (15.51)	104.91 (13.75)	<.001
Socioemotional Development	101.75 (14.86)	100.17 (15.23)	104.16 (13.95)	<.001
Motor Development	100.51 (15.03)	101.80 (15.57)	98.56 (13.97)	<.001
Total Development	101.86 (14.83)	100.40 (15.07)	104.06 (14.16)	<.001

^a^ All *P* values were corrected for multiple testing using Holm Bonferroni procedure.

^b^ Body mass index is calculated as weight in kilograms divided by height in meters squared.

There were differences between children by urbanicity. Children from rural areas had a higher prevalence of stunting (1435 children [33.74%] vs 483 children [17.16%]) and wasting (602 children [14.37%] vs 344 children [12.61%]), smaller mid–upper arm fat area (6.50 cm^2 ^vs 6.92 cm^2^) and lean area (14.77 cm^2 ^vs 15.44 cm^2^), and lower overall child development score (100.40 vs 104.06, a difference of 4 points or approximately one-fourth of 1 SD) than those from urban areas. Children in urban areas performed better in all domains of the EAP-ECDS except motor development, in which they performed worse with a 3-point (one-fifth of 1 SD) difference.

Table 2 shows the prevalence data of the 5 countries after weighting for age, sex, and urbanicity. The prevalence of stunting was highest in Papua New Guinea (55.0%), followed by Vanuatu (45.8%) and Cambodia (36.9%). The prevalence of wasting was the highest in Cambodia (35.9%), followed by Vanuatu (12.8%) and Papua New Guinea (7.7%). Meanwhile, the prevalence of overweight was 53.7% in Vanuatu and 29.8% in Papua New Guinea.

**Table 2.** Prevalence of Malnutrition Among Children Aged 3 to 5 Years From Cambodia, China, Mongolia, Papua New Guinea, and Vanuatu

Measure of malnutrition and country	Children, No.	Children, weighted No. (95% CI)^a^^,^^b^	Children, weighted % (95% CI)^b^
Stunting (height-for-age *z *score less than −2)			
East Asia			
China	47 722	559.00 (320.73-797.26)	1.2 (0.67-1.67)
Mongolia	177	18.19 (15.22-21.16)	10.3 (8.60-11.96)
Southeast Asia and Pacific			
Cambodia	1033	381.16 (355.96-406.37)	36.9 (34.46-39.34)
Papua New Guinea	580	319.08 (305.75-332.40)	55.0 (52.72-57.31)
Vanuatu	20	9.15 (8.47-9.84)	45.8 (42.34-49.19)
Wasting (weight-for-height *z *score less than −2)			
East Asia			
China	47 722	3173.64 (2621.88-3725.39)	6.7 (5.49-7.81)
Mongolia	177	9.49 (7.28-11.69)	5.4 (4.11-6.61)
Southeast Asia and Pacific			
Cambodia	1033	370.79 (345.73-395.85)	35.9 (33.47-38.32)
Papua New Guinea	580	44.66 (37.52-51.81)	7.7 (6.47-8.93)
Vanuatu	20	2.55 (2.10-3.01)	12.8 (10.48-15.07)
Overweight (weight-for-height *z *score greater than 2)			
East Asia			
China	47 722	3024.92 (2485.35-3564.48)	6.3 (5.21-7.47)
Mongolia	177	11.76 (9.32-14.20)	6.6 (5.27-8.02)
Southeast Asia and Pacific			
Cambodia	1033	22.26 (14.68-29.85)	2.2 (1.42-2.89)
Papua New Guinea	580	173.01 (160.75-185.27)	29.8 (27.72-31.94)
Vanuatu	20	10.74 (10.05-11.42)	53.7 (50.26-57.11)

^a^ Numbers are shown in 1000s.^16^

^b^ Estimates are weighted on age, sex, and urbanicity.

The associations of nutritional status and body composition with total development are shown in eFigure 1 in the Supplement. HFA, mid–upper AC, and lean area were linearly associated with total development after adjusting for age, sex, urbanicity, and family SES index. The association between fat proportion and total development was nonlinear. Higher fat proportion *z* score was associated lower total development until the *z* score was greater than 0, after which the association appeared to be null. Similar patterns of association were found for the Cognitive, Language/Emergent Literary, Socioemotional, and Motor Development subscales (eFigure 2, eFigure 3, eFigure 4, eFigure 5, and eFigure 6 in the Supplement) with some exceptions.

**Figure 1.**
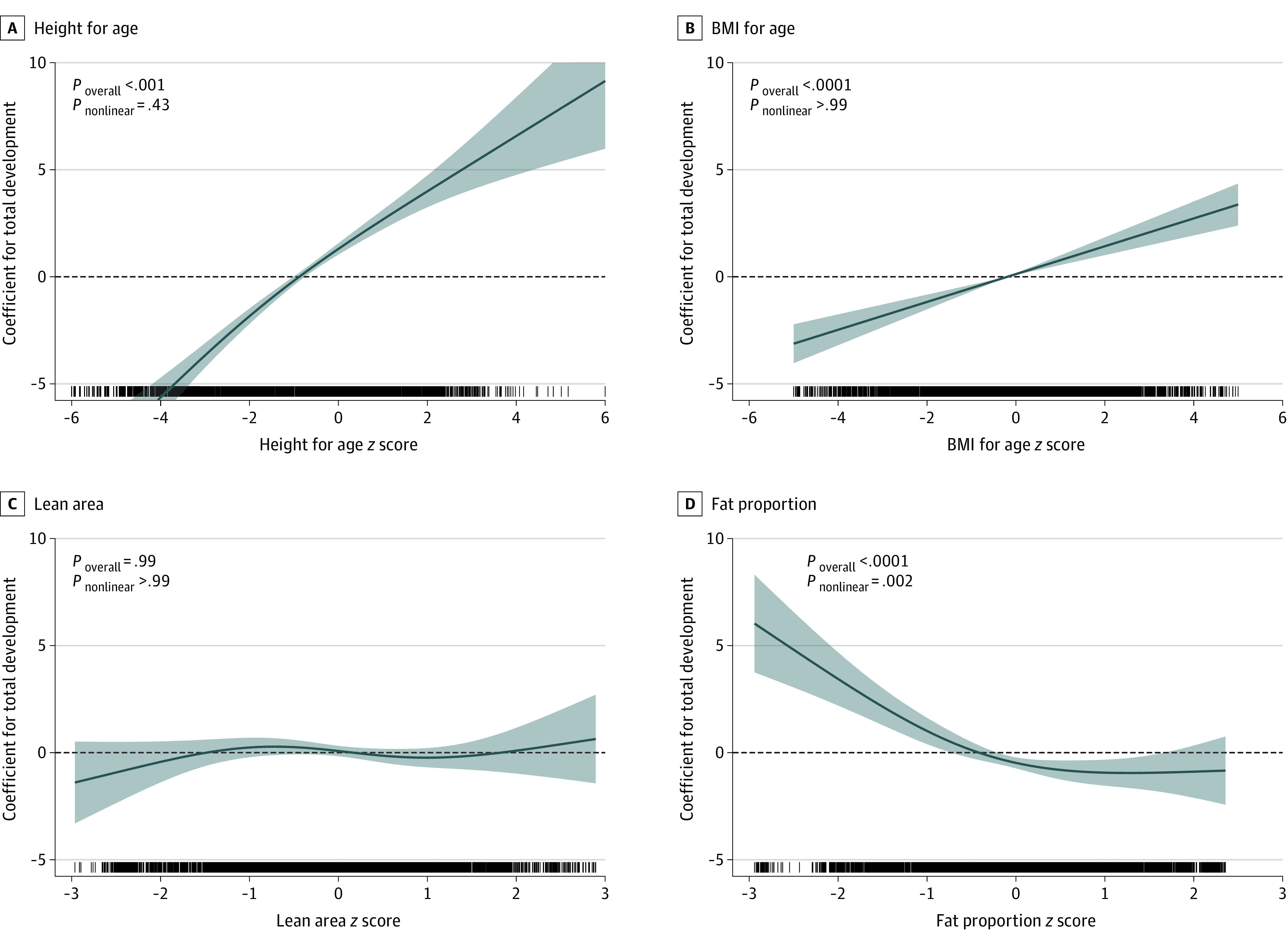
Nonlinear Association of Nutritional Status and Body Composition With Total Development Data were adjusted for all included nutritional status and body composition indicators, as well as age in months, sex, urbanicity, and family socioeconomic index; country and provinces were modeled as random intercepts. Lines denote coefficients, and shaded areas denote 95% CIs. *P* < .006 is regarded as significant according to Bonferroni criteria. BMI indicates body mass index (calculated as weight in kilograms divided by height in meters squared).

HFA, BMI-for-age (instead of WFH), lean area (instead of AC, TS, and fat area), and fat proportion were selected for fully adjusted models on the basis of higher *R*^2^ compared with their counterparts. Their independent nonlinear associations with child development are shown in Figure 1. HFA and BMI-for-age were linearly associated with Total and the domain-specific development. Low (*z* score less than −1) lean area was associated with lower Motor Development scores but not with other outcomes. Low (*z* score less than −1) fat proportion was associated with better scores on the Total, Cognitive, Language/Emergent Literacy, and Motor Development subscales. On average, each unit increase in *z* score was associated with an increase in points on the Total Development domain for HFA (β, 1.57; 95% CI, 1.35-1.80) and BMI-for-age (β, 0.64; 95% CI, 0.45-0.82) (Table 3). After adjustment for BMI and height, better ECD was associated with low body fat proportion (β, 0.93; 95% CI, 0.45-1.42). The β values were higher for Cognitive and Socioemotional development and lower for Language/Emergent Literacy. The results were consistent when country was modeled as a fixed factor (eTable 1 in the Supplement).

**Table 3.** Linear Association of Nutritional Status and Body Composition With Early Child Development

East Asia Pacific Early Child Development Scales subscale and factor	β (95% CI)^a^	*P* value^b^
Total Development		
Height-for-age	1.57 (1.35 to 1.80)	<.001
BMI-for-age	0.64 (0.45 to 0.82)	<.001
Lean area	−0.07 (−0.42 to 0.29)	.71
Fat proportion^c^	−0.93 (−1.42 to −0.45)	<.001
Cognitive Development		
Height-for-age	1.58 (1.34 to 1.82)	<.001
BMI-for-age	0.55 (0.36 to 0.75)	<.001
Lean area	−0.32 (−0.70 to 0.06)	.09
Fat proportion^c^	−0.68 (−1.20 to −0.16)	.02
Language/Emergent Literacy		
Height-for-age	1.35 (1.14 to 1.57)	<.001
BMI-for-age	0.46 (0.29 to 0.64)	<.001
Lean area	−0.14 (−0.48 to 0.21)	.87
Fat proportion	−0.07 (−0.54 to 0.39)	.87
Socioemotional Development		
Height-for-age	1.32 (1.07 to 1.57)	<.001
BMI-for-age	0.65 (0.45 to 0.85)	<.001
Lean area	−0.35 (−0.74 to 0.04)	.08
Fat proportion^c^	−1.46 (−2.00 to −0.92)	<.001
Motor Development		
Height-for-age	1.57 (1.28 to 1.87)	<.001
BMI-for-age	0.49 (0.25 to 0.73)	<.001
Lean area^c^	0.43 (−0.03 to 0.89)	.14
Fat proportion^c^	0.27 (−0.36 to 0.90)	.40

Abbreviation: BMI, body mass index (calculated as weight in kilograms divided by height in meters squared).

^a^ Adjusted for all included nutritional status and body composition indicators, as well as age in months, sex, urbanicity, and family socioeconomic status index; country and provinces were modeled as random intercepts.

^b^ *P* values were corrected for multiple testing using Holm Bonferroni procedure.

^c^ For evidence for nonlinear association, see Figure 1. These factors were selected according to results shown in Figure 1.

**Figure 2.**
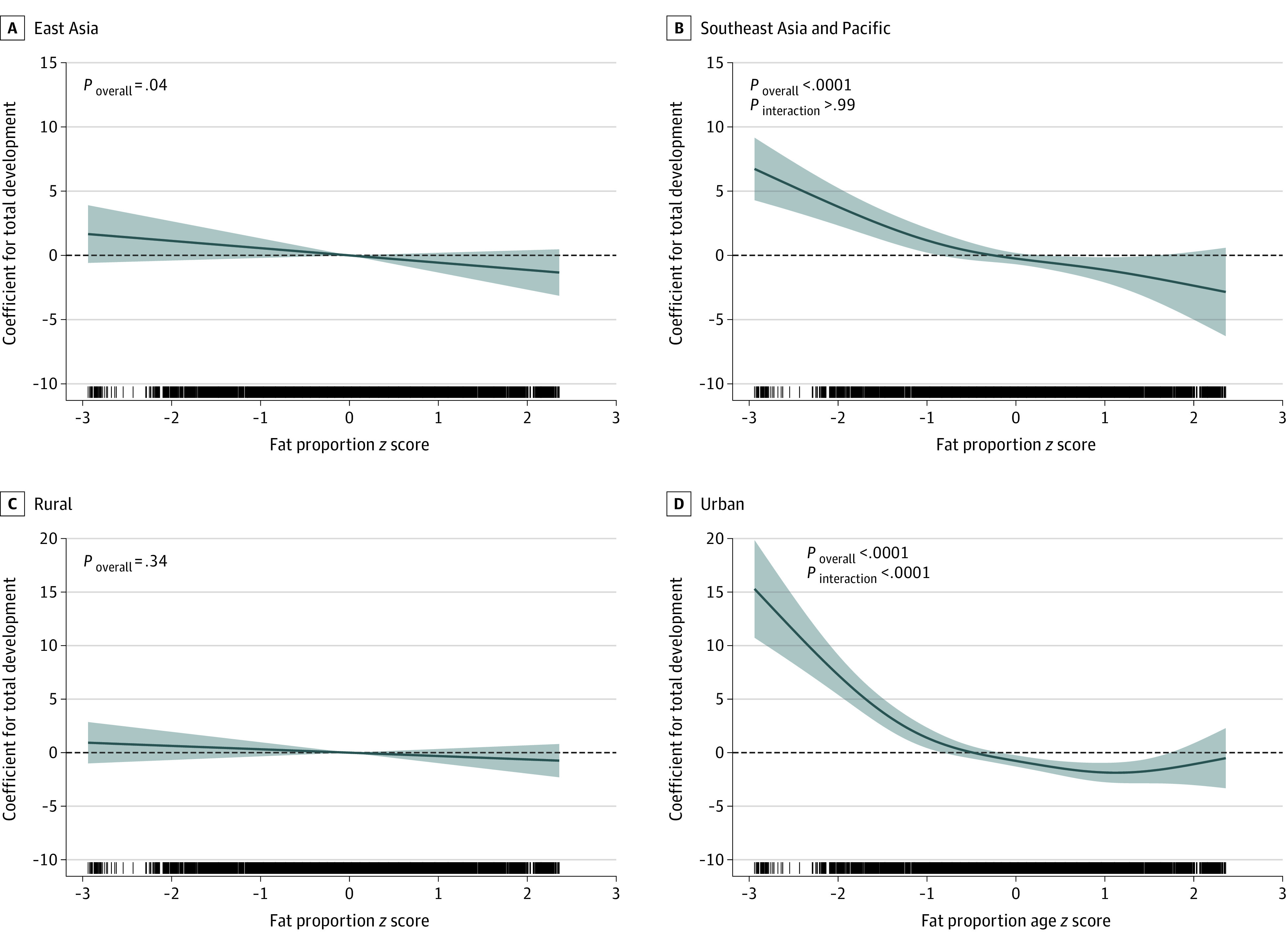
Nonlinear Association of Fat Proportion With Total Development by Region and Urbanicity Data were adjusted for height-for-age, body mass index (weight in kilograms divided by height in meters squared)–for-age, and lean mass *z *scores, as well as age in months, sex, urbanicity, and family socioeconomic status index; country and provinces were modeled as random intercepts. Lines denote coefficients, and shaded areas denote 95% CIs. *P* < .006 is regarded as significant according to Bonferroni criteria.

The associations with total development by region and urbanicity are shown in eTable 2 in the Supplement and in Figure 2. The associations were largely consistent across the subgroups, except that the β value for HFA was higher in East Asia (*P* for interaction = .002) and that of fat proportion was specific to children living in urban environment (*P* for interaction < .001).

## Discussion

This cross-sectional study examined the associations of nutritional status and body composition with ECD and found that HFA, BMI-for-age, and body fat proportion were independently associated with development. These findings indicate that in addition to linear growth, lean mass (represented by BMI adjusted for fat proportion) was also associated with ECD. The association of body fat proportion was more complex; after adjustment for overall mass and height, low body fat proportion was associated with better development but there was no difference between children with average and high fat proportion. To our knowledge, this is the first report on the nonlinear association between body fat proportion and ECD. Interestingly, fat stores (represented by fat area) were not associated with ECD.

Consistent with the findings of previous research, the present study has identified a significant association between linear growth and ECD. A recent meta-analysis of 68 studies^5^ has quantified that each SD increase in HFA was associated with a 0.09 SD increase in cognitive development and a 0.38 SD increase in motor development, assuming the associations were linear. This aggregated effect size of cognitive development was similar to what we found (β, 1.57, equivalent to 0.10 SD), but that of motor development was much larger than ours (β, 1.57, equivalent to 0.10 SD). The inconsistency of motor development’s effect size could be attributed to the small sample size of the meta-analysis (only 2 studies in that analysis), as well as the age difference (both studies recruited children younger than 2 years). In addition to cognitive and motor development, the present study provided new evidence for the association between linear growth and socioemotional development, which is at least as important as cognitive development for long-term achievement.^19^

Unlike body height, there seems to be a lack in systematic reviews and meta-analyses regarding the role of wasting, WFH, and BMI-for-age on ECD. In fact, previous estimates of developmental vulnerabilities only used stunting as a proxy but not wasting or weight-related factors.^4^ Given that BMI-for-age, independent of linear growth, was significantly associated with suboptimal development, future estimation of developmental issues should consider both the children’s height and weight for a more comprehensive picture.

Nonetheless, we should note that although we identified a linear association between BMI-for-age and development in the fully adjusted model, this does not imply that overweight children would be better developed. The results were adjusted for body fat proportion, and BMI-for-age should therefore be interpreted as lean mass, rather than overall weight. In fact, when fat proportion was not adjusted, BMI-for-age was not associated with development.

There are multiple explanations for the associations between growth parameters and ECD. First, growth restriction may be an indicator of nutritional deficiencies, which are the potentially true cause of developmental issues. Early childhood is a critical period for brain development, which requires appropriate amount of nutrients.^20^ This nutritional hypothesis is also supported by previous a meta-analysis of the association of nutrient supplementation interventions with ECD.^20^

Second, both stunting and delayed ECD could be indicators of underlying medical factors, such as chronic or recurring infections.^21^ In low- and middle-income countries where clean water and sanitation are scarce, chronic infection is common.^22^ Similarly, various intrauterine exposures relating to poverty such as maternal infection, undernutrition, and insufficient antenatal care can also cause intrauterine growth retardation,^23^ which is associated with both smaller body size and subpar ECD.^24^

Third, growth restriction may be an indicator for poverty and insufficient environmental stimulation. Environmental stimulation is necessary for optimal ECD because it helps to form and refine the neuronal connectivity of young children.^25^ The constraints of poverty and challenging environments all too often result in low levels of parental involvement, and these may result in both undernutrition and understimulation. Undernourished children have been shown to be less likely to learn on their own^26^ and their parents to be less responsive and caring.^27^ It should also be noted that preschool attendance is associated with better ECD regardless of stunting status and, therefore, should be scaled up to improve ECD.^28^

This study found that after adjusting for their body height and weight, children in an urban setting with lower fat proportion had better rather than worse development, but that body fat proportion was otherwise not associated with ECD. This suggests that acute undernutrition, as reflected in reduced fat stores, is not associated with the risk of delayed ECD in this East Asian and Pacific population and that any association between low body weight and ECD reflects variance in lean rather than fat mass. Whether this is a general observation or one specific to these ethnic groups is pending further studies in other regions.

It is not clear why lower fat proportion was associated with higher ECD in urban settings, but it might an indicator of being more physically active. Body fatness is correlated with physical activity level,^29^ and extensive clinical studies have shown that exercise could improve cognitive ability.^30^ Individuals who are more active were found to have increased frequency in the δ, θ, and β spectral bands in the electroencephalograms.^31^ Animal studies have also found that physical activity could lead to better hippocampal cell proliferation and survival.^32^ Further studies measuring physical activity level will be needed to verify this hypothesis.

### Limitations

There are several limitations to this study. First, this is a cross-sectional study; therefore, causality could not be ascertained. In fact, we hypothesize that growth and body composition parameters could be risk markers for ECD. Second, there is no standard reference for body composition parameters, which limited us from providing the relative condition of fat area, lean area, and fat proportion of children in the EAPR. Third, the associations between nutritional parameters and development were only modest, indicating that it is impractical to tackle underdevelopment using only a nutritional approach. Health, education, and family characteristics will be needed to accurately reflect the ECD of children.

## Conclusions

This study found that HFA, BMI-for-age, and body fat proportion were independently associated with development. Future studies should consider using these parameters to estimate the prevalence of underdevelopment. Nutritional trials should investigate to what extent these associations are causal.

## Notes

Supplement. **eTable 1.** Sensitivity Analysis on the Linear Association of Nutritional Status and Body Composition With Child Development **eTable 2.** Linear Association of Nutritional Status and Body Composition With Total Development by Region and Urbanicity **eFigure 1.** Nonlinear Association of Nutritional Status and Body Composition With Total Development **eFigure 2.** Nonlinear Association of Nutritional Status and Body Composition With Cognitive Development **eFigure 3.** Nonlinear Association of Nutritional Status and Body Composition With Language and Emergent Literacy Development **eFigure 4.** Nonlinear Association of Nutritional Status and Body Composition With Socioemotional Development **eFigure 5.** Nonlinear Association of Nutritional Status and Body Composition With Motor Development **eFigure 6.** Nonlinear Association of Nutritional Status and Body Composition With Total Development by Region and Urbanicity

## References

[1] Black RE, Allen LH, Bhutta ZA, ; Maternal and Child Undernutrition Study Group. Maternal and child undernutrition: global and regional exposures and health consequences. Lancet. 2008;371(9608):243-260. doi:10.1016/S0140-6736(07)61690-0 18207566

[2] de Onís M, Monteiro C, Akré J, Glugston G. The worldwide magnitude of protein-energy malnutrition: an overview from the WHO Global Database on Child Growth. Bull World Health Organ. 1993;71(6):703-712. 8313488 PMC2393544

[3] UNICEF. Statistical Tables: The State of the World’s Children 2017. UNICEF; 2018.

[4] Grantham-McGregor S, Cheung YB, Cueto S, Glewwe P, Richter L, Strupp B; International Child Development Steering Group. Developmental potential in the first 5 years for children in developing countries. Lancet. 2007;369(9555):60-70. doi:10.1016/S0140-6736(07)60032-4 17208643 PMC2270351

[5] Sudfeld CR, McCoy DC, Danaei G, . Linear growth and child development in low- and middle-income countries: a meta-analysis. Pediatrics. 2015;135(5):e1266-e1275. doi:10.1542/peds.2014-3111 25847806

[6] Munafò MR, Tilling K, Taylor AE, Evans DM, Davey Smith G. Collider scope: when selection bias can substantially influence observed associations. Int J Epidemiol. 2018;47(1):226-235. doi:10.1093/ije/dyx206 29040562 PMC5837306

[7] Webb KE, Horton NJ, Katz DL. Parental IQ and cognitive development of malnourished Indonesian children. Eur J Clin Nutr. 2005;59(4):618-620. doi:10.1038/sj.ejcn.1602103 15688080

[8] Corsi DJ, Subramanyam MA, Subramanian SV. Commentary: measuring nutritional status of children. Int J Epidemiol. 2011;40(4):1030-1036. doi:10.1093/ije/dyr108 21724577

[9] Asia-Pacific Regional Network for Early Childhood. Validation, finalization and adoption of the East Asia-Pacific Early Child Development Scales (EAP-ECDS). Accessed November 12, 2021. https://arnec.net/publication/full-report:-east-asia-pacific-early-child-development-scales-(eap-ecds)

[10] de Onis M, Onyango AW, Van den Broeck J, Chumlea WC, Martorell R. Measurement and standardization protocols for anthropometry used in the construction of a new international growth reference. Food Nutr Bull. 2004;25(1)(suppl):S27-S36. doi:10.1177/15648265040251S105 15069917

[11] WHO Multicentre Growth Reference Study Group. WHO Child Growth Standards based on length/height, weight and age. Acta Paediatr Suppl. 2006;450(S450):76-85. doi:10.1111/j.1651-2227.2006.tb02378.x 16817681

[12] Rolland-Cachera MF, Brambilla P, Manzoni P, . Body composition assessed on the basis of arm circumference and triceps skinfold thickness: a new index validated in children by magnetic resonance imaging. Am J Clin Nutr. 1997;65(6):1709-1713. doi:10.1093/ajcn/65.6.1709 9174464

[13] Rao N, Sun J, Richards B, . Assessing diversity in early childhood development in the East Asia-Pacific. Child Indicators Res. 2019;12(1):235-254. doi:10.1007/s12187-018-9528-5

[14] Rao N, Sun J, Ng M, . Validation, Finalization and Adoption of the East Asia-Pacific Early Child Development Scales (EAP-ECDS). UNICEF; 2014.

[15] Vyas S, Kumaranayake L. Constructing socio-economic status indices: how to use principal components analysis. Health Policy Plan. 2006;21(6):459-468. doi:10.1093/heapol/czl029 17030551

[16] United Nations Department of Economic and Social Affairs Population Division. World Population Prospects: The 2015 Revision. United Nations; 2015.

[17] Wood SN. Generalized Additive Models: An Introduction. R. Chapman and Hall/CRC Press; 2017.

[18] Ruppert D. Selecting the number of knots for penalized splines. J Comput Graph Stat. 2002;11(4):735-757. doi:10.1198/106186002853

[19] Heckman JJ. Skill formation and the economics of investing in disadvantaged children. Science. 2006;312(5782):1900-1902. doi:10.1126/science.1128898 16809525

[20] Ip P, Ho FKW, Rao N, . Impact of nutritional supplements on cognitive development of children in developing countries: a meta-analysis. Sci Rep. 2017;7(1):10611. doi:10.1038/s41598-017-11023-4 28878390 PMC5587553

[21] Wright C, Garcia AL. Too much effort for too little effect: time to reconsider the merits of food supplementation programs? J Nutr. 2020;150(2):190-191. doi:10.1093/jn/nxz304 31848602

[22] Guerrant RL, DeBoer MD, Moore SR, Scharf RJ, Lima AA. The impoverished gut: a triple burden of diarrhoea, stunting and chronic disease. Nat Rev Gastroenterol Hepatol. 2013;10(4):220-229. doi:10.1038/nrgastro.2012.239 23229327 PMC3617052

[23] Romo A, Carceller R, Tobajas J. Intrauterine growth retardation (IUGR): epidemiology and etiology. Pediatr Endocrinol Rev. 2009;6(3)(suppl):332-336. 19404231

[24] Fattal-Valevski A, Toledano-Alhadef H, Leitner Y, Geva R, Eshel R, Harel S. Growth patterns in children with intrauterine growth retardation and their correlation to neurocognitive development. J Child Neurol. 2009;24(7):846-851. doi:10.1177/0883073808331082 19617460

[25] Phillips DA, Shonkoff JP. From Neurons to Neighborhoods: The Science of Early Childhood Development. National Academies Press; 2000. 25077268

[26] Strupp BJ, Levitsky DA. Enduring cognitive effects of early malnutrition: a theoretical reappraisal. J Nutr. 1995;125(8)(suppl):2221S-2232S. doi:10.1093/jn/125.suppl_8.2221S 7542704

[27] Wachs TD, Georgieff M, Cusick S, McEwen BS. Issues in the timing of integrated early interventions: contributions from nutrition, neuroscience, and psychological research. Ann N Y Acad Sci. 2014;1308(1):89-106. doi:10.1111/nyas.12314 24354763 PMC4075015

[28] Rao N, Richards B, Lau C, . Associations among early stimulation, stunting, and child development in four countries in the East Asia–Pacific. IJEC. 2020;52:175-193. doi:10.1007/s13158-020-00270-8

[29] Moore LL, Nguyen U-SD, Rothman KJ, Cupples LA, Ellison RC. Preschool physical activity level and change in body fatness in young children: the Framingham Children’s Study. Am J Epidemiol. 1995;142(9):982-988. doi:10.1093/oxfordjournals.aje.a117747 7572980

[30] Hillman CH, Erickson KI, Kramer AF. Be smart, exercise your heart: exercise effects on brain and cognition. Nat Rev Neurosci. 2008;9(1):58-65. doi:10.1038/nrn2298 18094706

[31] Lardon MT, Polich J. EEG changes from long-term physical exercise. Biol Psychol. 1996;44(1):19-30. doi:10.1016/S0301-0511(96)05198-8 8906355

[32] Brown J, Cooper-Kuhn CM, Kempermann G, . Enriched environment and physical activity stimulate hippocampal but not olfactory bulb neurogenesis. Eur J Neurosci. 2003;17(10):2042-2046. doi:10.1046/j.1460-9568.2003.02647.x 12786970

