# Supplementary material for: Association of Early Nutritional Status With Child Development in the Asia Pacific Region
Source: JAMA Netw Open. 2021 Dec 16;4(12):e2139543. doi: 10.1001/jamanetworkopen.2021.39543 (PMC8678697; doi:10.1001/jamanetworkopen.2021.39543)
Supplement: Supplement. — eTable 1. Sensitivity Analysis on the Linear Association of Nutritional Status and Body Composition With Child Development eTable 2. Linear Association of Nutritional Status and Body Composition With Total Development by Region and Urbanicity eFigure 1. Nonlinear Association of Nutritional Status and Body Composition With Total Development eFigure 2. Nonlinear Association of Nutritional Status and Body Composition With Cognitive Development eFigure 3. Nonlinear Association of Nutritional Status and Body Composition With Language and Emergent Literacy Development eFigure 4. Nonlinear Association of Nutritional Status and Body Composition With Socioemotional Development eFigure 5. Nonlinear Association of Nutritional Status and Body Composition With Motor Development eFigure 6. Nonlinear Association of Nutritional Status and Body Composition With Total Development by Region and Urbanicity [file jamanetwopen-e2139543-s001.pdf]

Supplemental Online Content

Ho FK, Rao N, Tung KTS, et al. Association of early nutritional status with child development in the Asia Pacific region. *JAMA Netw Open*. 2021;4(12):e2139543. doi:10.1001/jamanetworkopen.2021.39543

eTable 1. Sensitivity Analysis on the Linear Association of Nutritional Status and Body Composition With Child Development

eTable 2. Linear Association of Nutritional Status and Body Composition With Total Development by Region and Urbanicity

eFigure 1. Nonlinear Association of Nutritional Status and Body Composition With Total Development

eFigure 2. Nonlinear Association of Nutritional Status and Body Composition With Cognitive Development

eFigure 3. Nonlinear Association of Nutritional Status and Body Composition With Language and Emergent Literacy Development

eFigure 4. Nonlinear Association of Nutritional Status and Body Composition With Socioemotional Development

eFigure 5. Nonlinear Association of Nutritional Status and Body Composition With Motor Development

eFigure 6. Nonlinear Association of Nutritional Status and Body Composition With Total Development by Region and Urbanicity

This supplemental material has been provided by the authors to give readers additional information about their work.

© 2021 Ho FK et al. *JAMA Network Open.*

eTable 1. Sensitivity Analysis on the Linear Association of Nutritional Status and Body Composition With Child Development

	β (95% CI)	P
**Total development**		
Height-for-age	1.57 (1.34, 1.80)	< .001
BMI-for-age	0.64 (0.45, 0.82)	< .001
Muscle area	-0.07 (-0.42, 0.29)	0.71
Fat proportion *	-0.94 (-1.43, -0.45)	< .001
**Cognitive development**		
Height-for-age	1.57 (1.33, 1.82)	< .001
BMI-for-age	0.56 (0.36, 0.75)	< .001
Muscle area	-0.32 (-0.70, 0.05)	.09
Fat proportion *	-0.69 (-1.21, -0.17)	.02
**Language/emergent literacy**		
Height-for-age	1.35 (1.13, 1.57)	< .001
BMI-for-age	0.46 (0.29, 0.64)	< .001
Muscle area	-0.14 (-0.48, 0.20)	0.85
Fat proportion	-0.09 (-0.56, 0.38)	0.85
**Socioemotional development**		
Height-for-age	1.31 (1.06, 1.56)	< .001
BMI-for-age	0.65 (0.45, 0.85)	< .001
Muscle area	-0.35 (-0.74, 0.04)	0.13
Fat proportion *	-1.47 (-2.01, -0.93)	< .001
**Motor development**		
Height-for-age	1.58 (1.29, 1.88)	< .001
BMI-for-age	0.50 (0.26, 0.74)	< .001
Muscle area *	0.44 (-0.02, 0.90)	.30
Fat proportion *	0.31 (-0.32, 0.94)	> .99

These factors were selected based on results shown in Figure 1.
Adjusted for all included nutritional status and body composition indicators, as well as age in months, sex, urbanicity, family SES index and country; provinces were modelled as random intercepts.
P-values were corrected for multiple testing using Holm Bonferroni procedure.
*Evidence for nonlinear association (see Figure 1)

© 2021 Ho FK et al. *JAMA Network Open.*

eTable 2. Linear Association of Nutritional Status and Body Composition With Total Development by Region and Urbanicity

	**Region**				**Urbanicity**			
	**East Asia**		**Southeast Asia & Pacific**		**Rural**		**Urban**	
	**β (95% CI)**	**P**	**β (95% CI)**	**P**	**β (95% CI)**	**P**	**β (95% CI)**	**P**
Height-for-age	1.10 (0.78, 1.41)	< .001	1.91 (1.58, 2.24)	< .001	1.58 (1.26, 1.91)	< .001	1.52 (1.20, 1.83)	< .001
BMI-for-age	0.58 (0.32, 0.84)	< .001	0.74 (0.48, 1.00)	< .001	0.48 (0.22, 0.74)	< .001	0.72 (0.47, 0.98)	< .001
Lean area	-0.55 (-1.10, 0.00)	.10	0.26 (-0.23, 0.74)	.60	0.20 (-0.32, 0.73)	.89	-0.13 (-0.61, 0.35)	.60
Fat proportion *	-0.61 (-1.24, 0.03)	.10	-1.60 (-2.38, -0.81)	< .001	0.18 (-0.57, 0.93)	.89	-1.81 (-2.48, -1.13)	< .001

These factors were selected based on results shown in Figure 1.
Adjusted for all included nutritional status and body composition indicators, as well as age in months, sex, urbanicity, and family SES index; country and provinces were modelled as random intercepts
*Evidence for nonlinear association (see Figure 2)

© 2021 Ho FK et al. *JAMA Network Open.*

eFigure 1. Nonlinear Association of Nutritional Status and Body Composition With Total Development

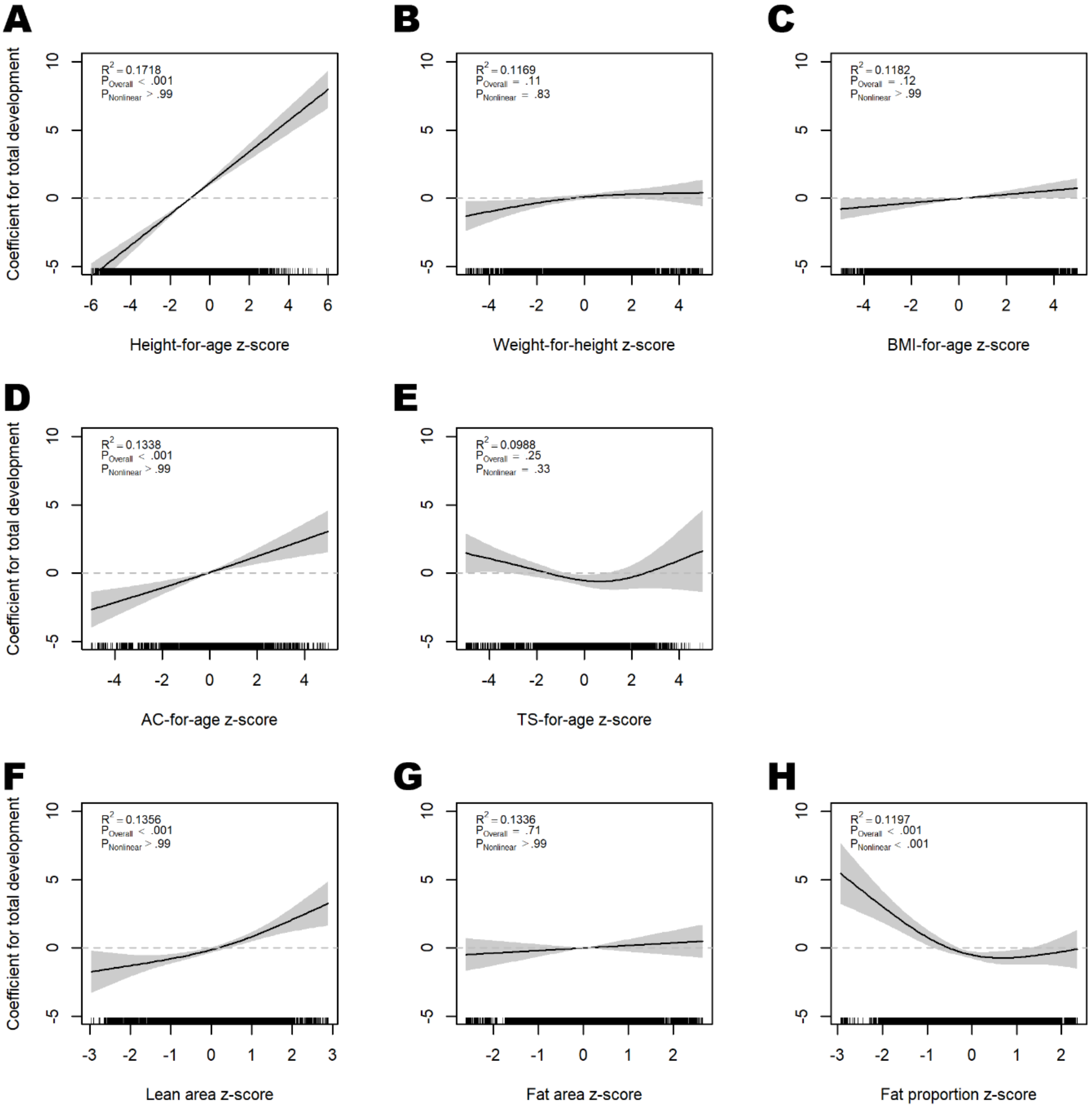

Not mutually adjusted.
Adjusted for age in months, gender, urbanicity, and family SES index as covariates, and intra-class correlations of country and province as random intercepts.
P-values < 0.0055 are regarded as statistically significant based on Bonferroni criteria.
AC: mid-upper arm circumference; TS: triceps skinfold thickness

© 2021 Ho FK et al. *JAMA Network Open.*

eFigure 2. Nonlinear Association of Nutritional Status and Body Composition With Cognitive Development

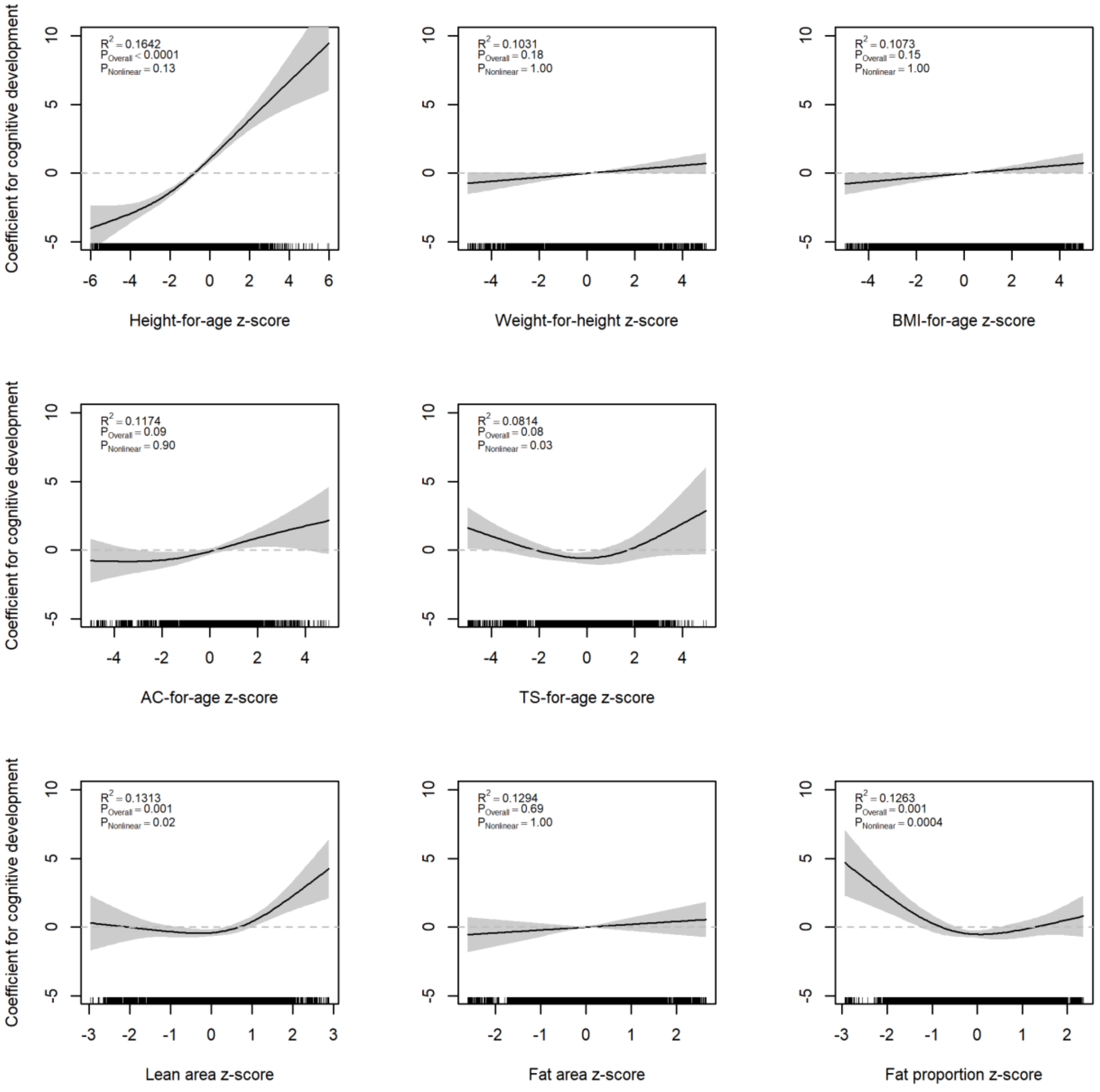

Not mutually adjusted.
Adjusted for age in months, gender, urbanicity, and family SES index as covariates, and intra-class correlations of country and province as random intercepts.
P-values < 0.0055 are regarded as statistically significant based on Bonferroni criteria.
AC: mid-upper arm circumference; TS: triceps skinfold thickness

© 2021 Ho FK et al. *JAMA Network Open.*

eFigure 3. Nonlinear Association of Nutritional Status and Body Composition With Language and Emergent Literacy Development

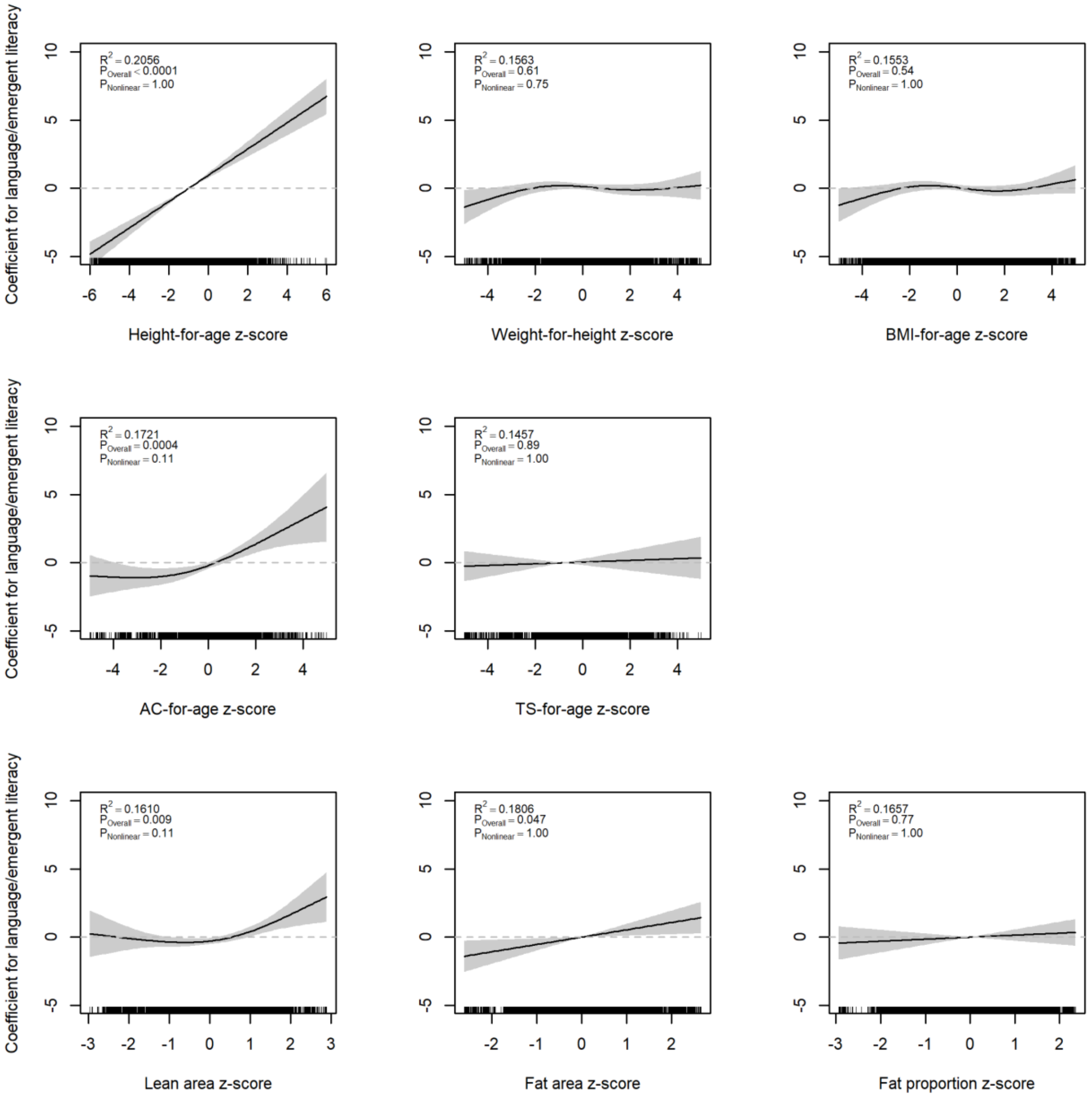

Not mutually adjusted.
Adjusted for age in months, gender, urbanicity, and family SES index as covariates, and intra-class correlations of country and province as random intercepts.
P-values < 0.006 are regarded as statistically significant based on Bonferroni criteria.
AC: mid-upper arm circumference; TS: triceps skinfold thickness

© 2021 Ho FK et al. *JAMA Network Open.*

eFigure 4. Nonlinear Association of Nutritional Status and Body Composition With Socioemotional Development

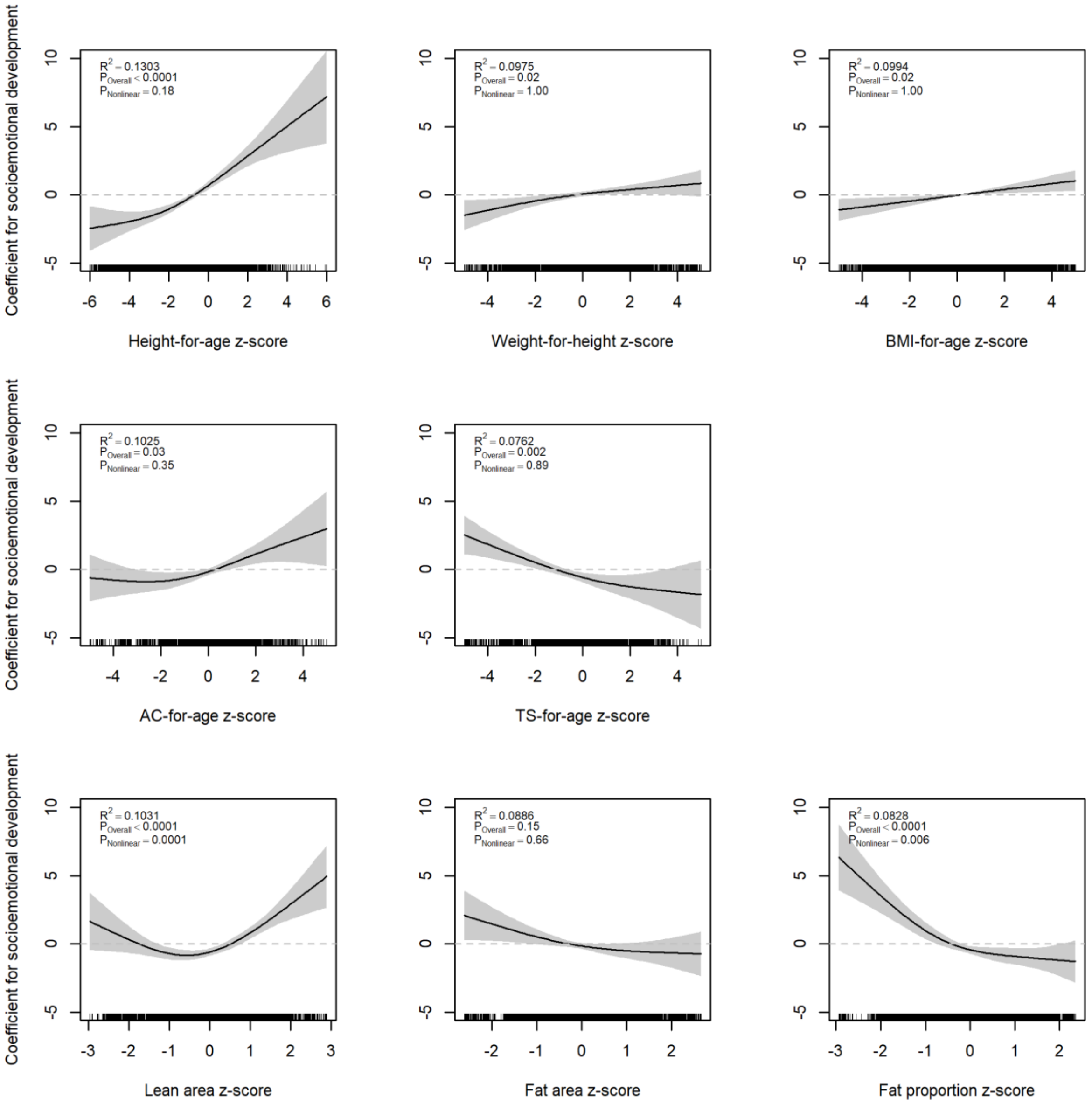

Not mutually adjusted.
Adjusted for age in months, gender, urbanicity, and family SES index as covariates, and intra-class correlations of country and province as random intercepts.
P-values < 0.006 are regarded as statistically significant based on Bonferroni criteria.
AC: mid-upper arm circumference; TS: triceps skinfold thickness

© 2021 Ho FK et al. *JAMA Network Open.*

eFigure 5. Nonlinear Association of Nutritional Status and Body Composition With Motor Development

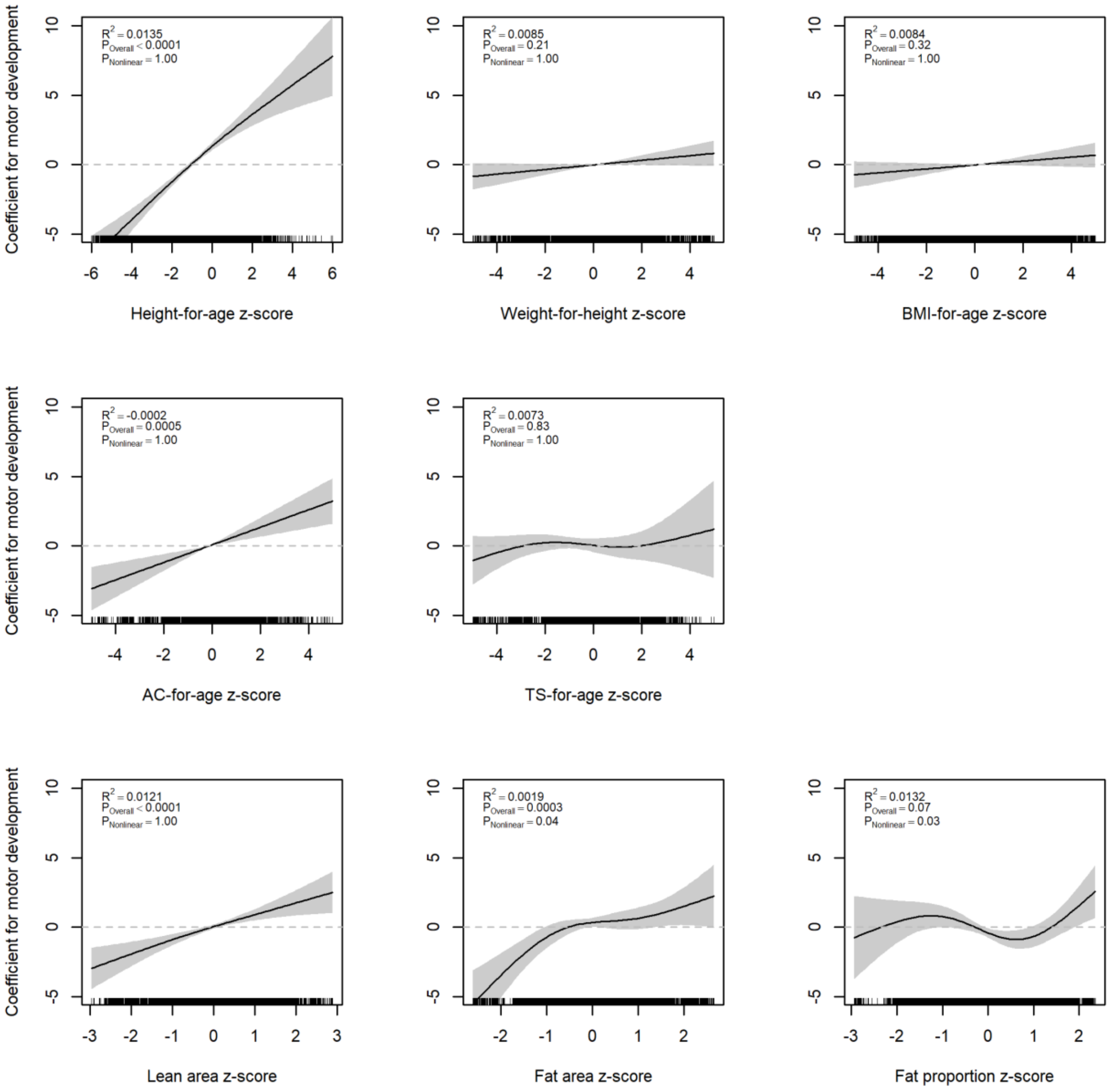

Not mutually adjusted.
Adjusted for age in months, gender, urbanicity, and family SES index as covariates, and intra-class correlations of country and province as random intercepts.
P-values < 0.006 are regarded as statistically significant based on Bonferroni criteria.
AC: mid-upper arm circumference; TS: triceps skinfold thickness

© 2021 Ho FK et al. *JAMA Network Open.*

eFigure 6. Nonlinear Association of Nutritional Status and Body Composition With Total Development by Region and Urbanicity

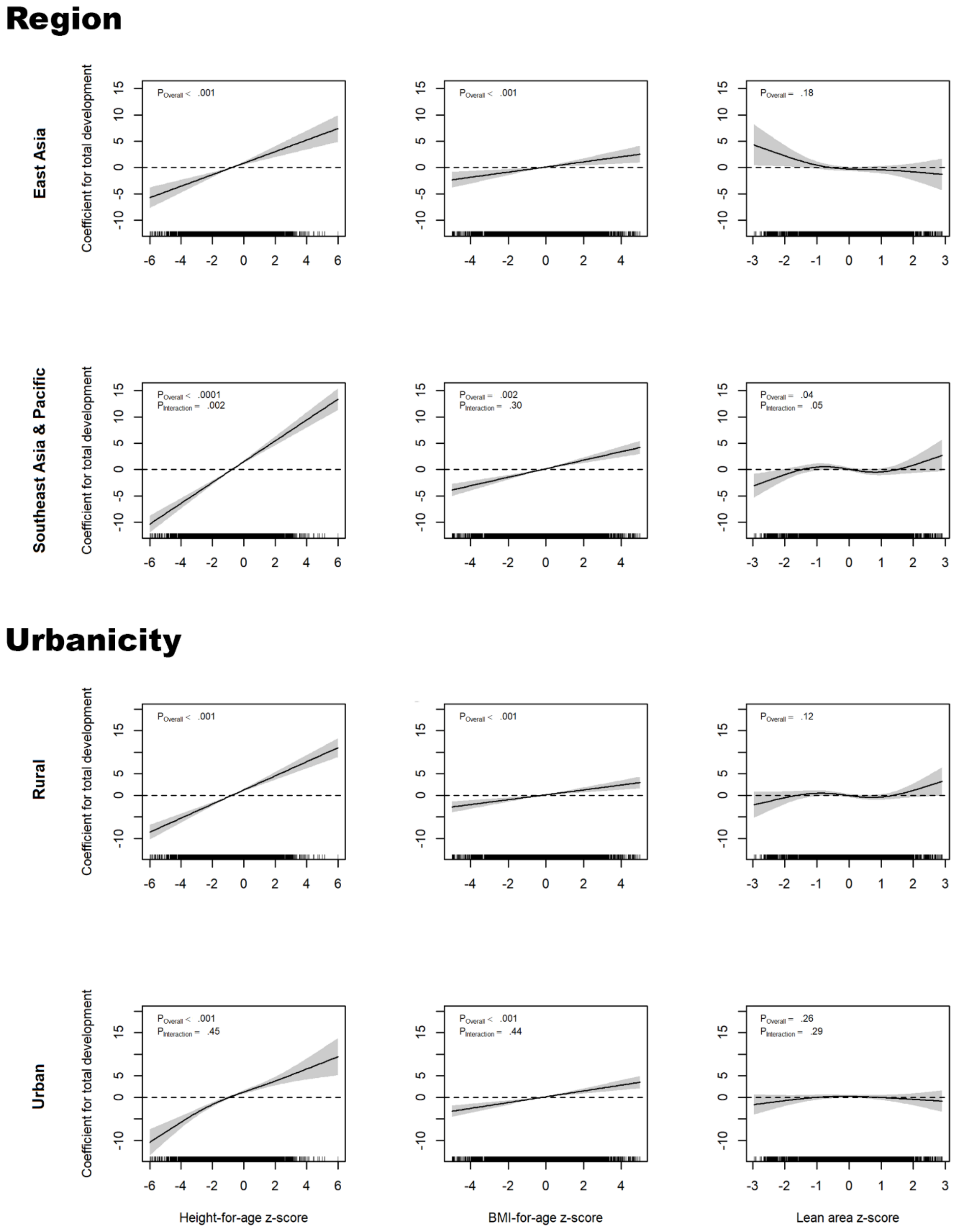

These factors were selected based on results shown in Figure 1. Adjusted for all included nutritional status and body composition indicators, as well as age in months, sex, urbanicity, and family SES index; country and provinces were modelled as random intercepts

P-values < .006 are regarded as statistically significant based on Bonferroni criteria.

© 2021 Ho FK et al. *JAMA Network Open.*
